# An evaluation of nine culturally tailored interventions designed to enhance engagement in HIV care among transgender women of colour in the United States

**DOI:** 10.1002/jia2.25991

**Published:** 2022-10-12

**Authors:** Gregory M. Rebchook, Deepalika Chakravarty, Jessica M. Xavier, JoAnne G. Keatley, Andres Maiorana, Jae Sevelius, Starley B. Shade, the SPNS Transgender Women of Color Study Group

**Affiliations:** ^1^ Division of Prevention Science Department of Medicine University of California San Francisco California USA; ^2^ Independent Consultant Silver Spring Maryland USA; ^3^ Innovative Response Globally for Transgender Women and HIV (IRGT) San Francisco California USA; ^4^ Department of Epidemiology and Biostatistics University of California San Francisco California USA

**Keywords:** transgender persons, HIV infections, delivery of healthcare, acquired immunodeficiency syndrome, continuity of patient care, evidence‐informed interventions

## Abstract

**Introduction:**

Transgender women (TW) worldwide have a high prevalence of HIV, and TW with HIV encounter numerous healthcare barriers. It is critical to develop evidence‐informed interventions to improve their engagement in healthcare to achieve durable viral suppression (VS). We evaluated whether participation in one of nine interventions designed specifically for TW was associated with improved engagement in HIV care among transgender women of colour (TWC).

**Methods:**

Between 2013 and 2017, nine US organizations implemented nine distinct and innovative HIV care engagement interventions with diverse strategies, including: individual and group sessions, case management and navigation, outreach, drop‐in spaces, peer support and/or incentives to engage TWC with HIV in care. The organizations enrolled 858 TWC, conducted surveys, captured intervention exposure data and extracted medical record data. Our evaluation of the interventions employed a pre‐post design and examined four outcomes—any HIV care visit, antiretroviral therapy (ART) prescription, retention in HIV care and VS (both overall and among those with a clinic visit and viral load test), at baseline and every 6 months for 24 months. We employed logistic generalized estimating equations to assess the relative odds of each outcome at 12 and 24 months compared to baseline.

**Results:**

Overall, 79% of participants were exposed to at least one intervention activity. Over 24 months of follow‐up, participants received services for a median of over 6 hours (range: 3–69 hours/participant). Compared to baseline, significantly (*p*<0.05) greater odds were demonstrated at both 12 and 24 months for three outcomes: prescription of ART (ORs: 1.42 at 12 months, 1.49 at 24 months), VS among all participants (ORs: 1.49, 1.54) and VS among those with a clinic visit and viral load test (ORs: 1.53, 1.98). The outcomes of any HIV care visit and retention in HIV care had significantly greater odds (ORs: 1.38 and 1.58, respectively) only at 12 months compared to baseline.

**Conclusions:**

These evaluation results illustrate promising approaches to improve engagement in HIV care and VS among TWC with HIV. Continued development, adaptation and scale‐up of culturally tailored HIV care interventions for this key population are necessary to meet the UNAIDS 95‐95‐95 goals.

## 1 INTRODUCTION

Transgender women (TW) are highly impacted by HIV worldwide and in the United States. Global HIV prevalence estimates among TW range from 19.9% to 40% [1, 2]. In the United States, HIV prevalence estimates among TW range from 14% to 42% [3, 4]. Transgender women of colour (TWC) have the highest prevalence of HIV in the United States, with 44.2% among African American TW and 25.8% among Latina TW [3].

Despite this disproportionate HIV burden, TW overall and TWC specifically have been overlooked in HIV surveillance, prevention, and treatment efforts. Until 2020, HIV surveillance in the United States did not separately monitor HIV incidence, prevalence, and mortality among TW [5]. Only a small number of prevention interventions, most developed in the past decade, have specifically addressed TWC [6, 7, 8, 9, 10, 11, 12]. In the absence of systematic national surveillance, the lack of a coordinated response to HIV disparities among TWC, and the ongoing epidemic of anti‐transgender violence [13, 14, 15], the devastating impact of HIV among TWC became a silent tragedy [16].

TWC with HIV face many barriers to engagement in HIV care and treatment. Intersectional stigmas [17], including transphobia, racism, discrimination, hostility, and provider bias, produce systemic barriers to care, such as poverty, homelessness, unemployment, and lack of insurance [18, 19, 20, 21]. The Ryan White HIV/AIDS Program (RWHAP), administered by the US Health Resources and Services Administration (HRSA), funds HIV care and treatment for low‐income persons. In 2020, about 1.8% of all RWHAP clients were TW [22]. Compared to their cisgender peers, TW RWHAP clients are more likely to be of colour, be younger, have unstable housing, and have incomes at or below the federal poverty level [22, 23]. They are also more likely to report unmet needs for food and nutrition, harm reduction, mental health, and housing services at entry into HIV care compared with cisgender women and men [24]. From 2016 to 2020, TW demonstrated retention in HIV care (range: 77.3–79.8%) close to the average for all RWHAP clients (79.4–81.7%), but they have notably lower than average viral suppression (VS; 79.0–84.2% vs. 84.9–89.4%) [22].

To address these disparities, the RWHAP funded a Special Program of National Significance (SPNS), *Enhancing Engagement and Retention in Quality HIV Care for Transgender Women of Color* in 2012. The RWHAP's SPNS Program has funded demonstration projects to improve HIV care engagement since 1991 [25]. SPNS interventions have pioneered behavioural and treatment approaches to improve access to and engagement in care and HIV service delivery for diverse populations [26, 27, 28, 29, 30, 31, 32, 33]. Until HRSA funded this initiative, there were no interventions focused upon improving TW's access to HIV primary care [34]. Nine demonstration projects were funded to develop and implement culturally tailored interventions to enhance engagement in HIV care among TWC with HIV. In this paper, we present the findings from the evaluation of health outcomes associated with these nine interventions.

## 2 METHODS

### 2.1 Settings and populations

The nine demonstration sites were located in four US urban centres: New York City, New York (Community Health Network in Queens and the State University of New York [SUNY]—Downstate in Brooklyn), Chicago, Illinois (Chicago House and Howard Brown Health Center), Los Angeles, California (Bienestar Human Services and Friends Research Institute) and California's San Francisco Bay area (Tri City Health Center in Fremont, Public Health Institute in Oakland and a partnership between the San Francisco Department of Health and the Asian/Pacific Islander Wellness Center in San Francisco). Additionally, HRSA funded the University of California, San Francisco to provide technical assistance and perform a multisite evaluation of the nine interventions.

### 2.2 Recruitment and eligibility

The sites used multiple recruitment and intervention strategies [35], including community outreach, social network recruitment, printed materials and referrals from clinics and other service providers to recruit participants into their interventions. To be eligible, participants had to be assigned male sex at birth; identify as transgender or female; and be at least 18 years old, living with HIV, and fluent in English or Spanish. Participants provided informed consent at enrolment. The study was approved annually by the Institutional Review Boards of each site and the evaluation centre.

### 2.3 Interventions

Intervention manuals are available that describe each of these nine interventions [36], and Table 1 provides an overview of the underlying theories and central components of each intervention. The interventions dynamically evolved over time—starting and stopping group sessions, adding new incentives, modifying modes of outreach, varying drop‐in hours, locations and policies—to meet participants’ needs.

**Table 1:** Intervention core elements and theories

*Intervention name*	TWEET [54]^a^	INFINI‐T [55]	Howard Brown^b^ [56]	Trans Life Care [57]	Brandy Martell Project [58]	Trans‐Access [58]	Princess Project [59]	Transactivate [60]	Alexis Project [61]
*Organization* *(sub‐population)*	Community Health Network	SUNY Downstate (adolescents and young adult TW)	Howard Brown Health Center	Chicago House	Tri City Health Center^c^	San Francisco Department of Public Health/Asian Pacific Islander Wellness Center^d^	Public Health Institute (Black/African American TW)	Bienestar Health Services (Latina TW)	Friends Research Institute
**Foundation for the intervention**									
	Social Cognitive Theory, Trans Theoretical Model	Singer's Syndemic Theory	Community‐tailored health intervention programmes that create safe spaces for transgender women	Trauma‐informed, Client centred, Strengths‐based, Systems theory, Harm reduction	Critical Race Theory	Trans‐affirming care, self‐actualizing services, mindful medicine, care coordination and continuity, harm reduction, community centred, radical healthcare	Motivational Enhancement Therapy, Motivational interviewing	Trans Theoretical Model, Strengths‐based perspective	Social Network Engagement, Social Cognitive Theory, Contingency Management/behavioural economics
**Intervention activities**									
Individual‐level services	Navigation by patient services specialist and retention specialist Assistance with benefits, name change, gender marker, referrals for trans‐affirming surgeries, referral to comprehensive legal services and trans‐sensitive shelters and housing specialists	Transgender peer youth advocates facilitated linkage and retention in HIV care Social work sessions—screening, referrals for mental health and substance use services. Case management	^e^	HIV care coordination/peer resource navigation On‐site medical services provided by a local clinic. Assistance with housing, legal and employment issues	Peer advocates and health educators provided navigation services Counselling and case management Direct legal counsel	Individualized, hands‐on support to clients to improve follow‐through on care plans Supplemental mental health support services Case manager to provide consistent support	Six Motivational Enhancement Intervention (MEI) sessions In‐hand referrals to needed services at partner agencies	Peer navigation to guide clients through Los Angeles's complex medical system and ensure engagement, re‐engagement and retention in HIV care. Motivational interviewing‐based linkage	Peer navigator to develop client‐centred treatment plans and link participants to healthcare and/or other services
Small group sessions	Series of five peer‐facilitated “teach back” groups focused on HIV/AIDS/STDs, sexual health, transitioning, wellness and mental health	One session of psychoeducational group	Weekly youth group and biweekly adult group	NA	16‐session workshop with five tracks	The first hour of weekly drop‐in clinic was devoted to a trans‐led support group	Weekly support group	NA	NA
Outreach (OR)	Weekly non‐traditional outreach and hosted social events	Worked with a community partner to provide outreach and engagement	Community outreach at trans events and venues	Event and street‐based OR	Social network recruitment; street, online and venue‐based OR; OR to referral agencies	OR to clients out of care or needing additional support (e.g. home visits)	Community, social network and online outreach and referral coupons	Social network testing, mobile testing, social network engagement, promotional materials to medical providers	Community‐wide social network recruitment, venue and street‐based OR, publicity materials, in‐reach and work with community partners to increase referrals
Drop‐in centre	NA	NA	A biweekly evening trans‐only drop in called “After Hours” that provided medical, pharmacy, needle exchange and behavioural healthcare, staff and community‐led programming, insurance counselling and dinner	Weekly Trans Safe resource drop‐in centre and on‐site medical services	NA	A weekly “Trans‐Access” clinic that provided comprehensive care and support services in a safe, welcoming and respectful setting	Butterfly nest: a safe space where participants and community members came to relax, take workshops and participate in support groups	NA	NA
Incentives offered to promote engagement in care, participation in activities or engaging peers in the intervention	Participants received small incentives for bringing in peers to the intervention	NA	NA	NA	Participants received a $50 gift card for each peer brought into the intervention. Participants received a $100 gift card.	NA	Participants received a $20 gift card for each successful referral (up to 3/participant) Participants received a $20 gift card for each MEI session Participants received a $50 gift card for completing clinic visits	Participants received a $50 gift card after their referred client was active in the programme for 6 months. Participants received a $10 gift card for enrolment	Escalating valued gift cards to incentivize both clinic attendance and achieving HIV milestones ($500 maximum for reaching all targeted HIV health‐promoting goals)

*Note*: Behind the scenes components (e.g. community trainings and community advisory boards) are not included as intervention components in this table.

^a^ References after each intervention name are their peer‐reviewed published results.

^b^ The intervention implemented by the Howard Brown Community Health Center did not have a stand‐alone identity. It was a combination of the After Hours Clinic and the TYRA and T‐Time support groups.

^c^ The Tri City Health Center is now called Bay Area Community Health.

^d^ The Asian Pacific Islander Wellness Center is now called the San Francisco Community Health Center.

^e^ Behavioural health, linkage to care, retention in care and case management services were all available on site at Howard Brown as part of its regular suite of services.

Abbreviations: MEI, Motivational Enhancement Intervention; NA, not applicable; OR, outreach; STDs, sexually transmitted diseases; TW, transgender women.

### 2.4 Evaluation

Data were collected from December 2013 to August 2017 from three sources.

#### 2.4.1 Participant survey

Prior to their first interaction with the intervention, participants were interviewed by non‐intervention staff or completed a self‐administered baseline survey in REDCap [37, 38] in the language of their choosing (English or Spanish). The survey included questions about demographic and psychosocial characteristics, as well as gender‐affirming and HIV care. Participants were paid $25–$40 for baseline survey completion.

#### 2.4.2 Medical chart data from electronic health systems

Sites that provided HIV care extracted medical chart data from all visits for all participants within each reporting period. Sites that did not provide HIV care obtained signed medical release forms from each participant and obtained medical record data from their HIV care providers. The 6 months prior to enrolment was treated as the baseline period. Data were also extracted for every succeeding 6‐month period through the end of the study.

#### 2.4.3 Intervention exposure data

Participants’ exposure to the intervention was recorded in real‐time by research staff at each site. Here, we use data from the first 24 months following enrolment.

### 2.5 Measures

*Participant survey* measures have been previously described [39]. In brief, demographic data included age, race/ethnicity, education, and financial insecurity in the past 6 months. Other baseline information categorized in a binary format (0 = No or missing, 1 = Yes) included homelessness in the past 6 months, exchange of sex to pay for necessities in the past 6 months, incarceration in the past 6 months, lack of transportation leading to a missed medical appointment in the past 12 months, presence of significant depression [40, 41, 42], gender‐based discrimination experienced in the past 6 months in employment and housing [43], ever experiencing transphobia [44], disclosure of transgender identity, disclosure of HIV status, and hormone use in the past 6 months. A healthcare empowerment score was also calculated (range: 1–5) [45].

*Intervention exposure data* included the date of exposure, length of exposure in minutes, and type(s) of contact (community outreach, individual face‐to‐face session, small group session, drop‐in centre or other individual virtual contact [e.g. telephone call, text message, email, social network site or postal mail]).

*Medical chart data from electronic health record systems* were used to create the following binary outcome variables (0 = No or missing, 1 = Yes):

- Any HIV care visit: The participant had an HIV outpatient ambulatory health services (OAHS) visit in the past 6 months.
- Antiretroviral therapy (ART) prescription: The participant had been prescribed ART in the past 6 months.
- Retention in HIV care: In the past 12 months, the participant had at least one OAHS visit in each 6‐month period with a minimum of 60 days between the first visit in the first 6‐month period and the last visit in the subsequent 6‐month period [46]. These calculated values were unavailable at baseline as we only recorded medical chart data during the 6‐month period preceding enrolment.
- VS: The participant had at least one HIV viral load test in the past 6 months and had a suppressed viral load (<200 copies/ml) at their last test in that period.
- VS among those with an OAHS visit and viral load test: Among participants who had both an OAHS visit and a viral load test in the past 6 months, the participants had a suppressed HIV viral load (<200 copies/ml) at their last test in the period. This variable represents the HRSA indicator for VS among RWHAP participants [46].

### 2.6 Analysis

Using information from baseline participant surveys, we computed descriptive statistics for the overall sample and by the site.

Using intervention exposure data, we assessed the proportion of participants who received any component of the intervention, the median and inter‐quartile range of the overall duration of interaction with the intervention, the proportion of participants who had each type of interaction (outreach, individual, group, drop‐in and virtual), and the median number of interactions of each type.

Using medical chart data, we computed the proportion of patients who had any HIV care visit, had an ART prescription, were retained in HIV care and had verification of VS (both overall and among those with an OAHS visit and viral load test) at baseline and each 6‐month period through 24 months of follow‐up. Using data from each site, we employed logistic generalized estimating equations (GEE) to assess the odds of each outcome at the 12‐ and 24‐month follow‐up intervals compared to baseline. These models included a linear variable for months‐since‐baseline (“period”) as well as a quadratic variable (“period”*“period”) for months‐since‐baseline‐squared to represent the curvilinear relationship between follow‐up time and the primary outcomes. We present unadjusted odds ratios and their 95% confidence intervals (CI). Next, we employed inverse probability of treatment weighting to assess whether the observed results were due to differences in participant characteristics at baseline. We employed logistic GEE models to estimate the probability of each participant being from their observed site given the observed distribution of participant characteristics across all sites. In these models, the sample for each site is compared to the total sample across all sites. These models included participant characteristics previously found to be associated with engagement in HIV care in our sample [39]. These control characteristics are listed in Table 2. We employed multiple imputation for missing data. Next, we used the results from each model (the predicted probability of being from each site among individuals from that site) to compute the weight for each individual from each site (weight = 1/predicted probability). This weight was then applied in a weighted GEE model as defined above. The result of these models is to create pseudo‐populations where the distribution of participant characteristics is similar across each site. These models have wider confidence limits compared to unweighted models as they employ re‐sampling (*n* = 100 iterations) to impute missing data and estimate weights. We present the adjusted odds ratios and their 95% CIs from these models in the Supplementary Materials. All analyses were performed in SAS 9.4 [47].

**Table 2:** Sample characteristics—overall and by demonstration site

	Overall	TWEET	INFINI‐T	Howard Brown	Trans Life Care	Trans Access	Brandy Martell Project	Princess Project	Transactivate	Alexis Project
	*n* (%)	*n* (%)	*n* (%)	*n* (%)	*n* (%)	*n* (%)	*n* (%)	*n* (%)	*n* (%)	*n* (%)
Race‐ethnicity										
Hispanic, Latina or of Spanish origin	417 (48.6%)	144 (88.9%)	11 (47.8%)	20 (19.2%)	6 (5.0%)	12 (22.2%)	14 (30.4%)	8 (13.3%)	150 (100%)	52 (37.4%)
Black, non‐Hispanic	364 (42.4%)	7 (4.3%)	11 (47.8%)	73 (70.2%)	113 (94.2%)	28 (51.9%)	32 (69.6%)	48 (80.0%)		52 (37.4%)
American Indian or Alaska Native, non‐Hispanic	9 (1.0%)									9 (6.5%)
Asian or Pacific Islander, non‐Hispanic	14 (1.6%)			2 (1.9%).		8 (14.8%)		1 (1.7%)		3 (2.2%)
Additional, non‐Hispanic	11 (1.3%)	3 (1.9%)								8 (5.8%)
Multiracial, non‐Hispanic	28 (3.3%)	1 (0.6%)		7 (6.7%)	1 (0.8%)	4 (7.4%)		3 (5.0%)		12 (8.6%)
No response	15 (1.7%)	7 (4.3%)	1 (4.3%)	2 (1.9%)		2 (3.7%)				3 (2.2%)
Education										
Less than grade 12	346 (40.3%)	79 (48.8%)	8 (34.8%)	22 (21.2%)	29 (24.2%)	14 (25.9%)	19 (41.3%)	23 (38.3%)	99 (66.0%)	53 (38.1%)
Completed grade 12	291 (33.9%)	39 (24.1%)	9 (39.1%)	34 (32.7%)	73 (60.8%)	24 (44.4%)	17 (37.0%)	17 (28.3%)	33 (22.0%)	45 (32.4%)
At least some college	195 (22.7%)	29 (17.9%)	6 (26.1%)	45 (43.3%)	18 (15.0%)	16 (29.6%)	9 (19.6%)	19 (31.7%)	14 (9.3%)	39 (28.1%)
No response	26 (3.0%)	15 (9.3%)		3 (2.9%)			1 (2.2%)	1 (1.7%)	4 (2.7%)	2 (1.4%)
Ran out of money for basics (past 6 months)?										
Yes	560 (65.3%)	100 (61.7%)	17 (73.9%)	72 (69.2%)	67 (55.8%)	33 (61.1%)	32 (69.6%)	38 (63.3%)	93 (62.0%)	108 (77.7%)
No	180 (21.0%)	44 (27.2%)	4 (17.4%)	16 (15.4%)	37 (30.8%)	11 (20.4%)	7 (15.2%)	2 (3.3%)	47 (31.3%)	12 (8.6%)
No response	118 (13.8%)	18 (11.1%)	2 (8.7%)	16 (15.4%)	16 (13.3%)	10 (18.5%)	7 (15.2%)	20 (33.3%)	10 (6.7%)	19 (13.7%)
Ever homeless (past 6 months)?										
Yes	352 (41.0%)	66 (40.7%)	8 (34.8%)	28 (26.9%)	69 (57.5%)	24 (44.4%)	19 (41.3%)	26 (43.3%)	39 (26.0%)	73 (52.5%)
No	381 (44.4%)	68 (42.0%)	13 (56.5%)	49 (47.1%)	35 (29.2%)	23 (42.6%)	24 (52.2%)	17 (28.3%)	104 (69.3%)	48 (34.5%)
No response	125 (14.6%)	28 (17.3%)	2 (8.7%)	27 (26.0%)	16 (13.3%)	7 (13.0%)	3 (6.5%)	17 (28.3%)	7 (4.7%)	18 (12.9%)
Exchanged sex to pay for necessities (past 6 months)?										
Yes	330 (38.5%)	59 (36.4%)	7 (30.4%)	23 (22.1%)	68 (56.7%)	25 (46.3%)	20 (43.5%)	29 (48.3%)	45 (30.0%)	54 (38.8%)
No	400 (46.6%)	75 (46.3%)	13 (56.5%)	53 (51.0%)	35 (29.2%)	24 (44.4%)	22 (47.8%)	17 (28.3%)	98 (65.3%)	63 (45.3%)
No response	128 (14.9%)	28 (17.3%)	3 (13.0%)	28 (26.9%)	17 (14.2%)	5 (9.3%)	4 (8.7%)	14 (23.3%)	7 (4.7%)	22 (15.8%)
Incarcerated (past 6 months)?										
Yes	83 (9.7%)	12 (7.4%)	2 (8.7%)	2 (1.9%)	10 (8.3%)	13 (24.1%)	6 (13.0%)	6 (10.0%)	13 (8.7%)	19 (13.7%)
No	665 (77.5%)	126 (77.8%)	18 (78.3%)	80 (76.9%)	94 (78.3%)	35 (64.8%)	39 (84.8%)	42 (70.0%)	134 (89.3%)	97 (69.8%)
No response	110 (12.8%)	24 (14.8%)	3 (13.0%)	22 (21.2%)	16 (13.3%)	6 (11.1%)	1 (2.2%)	12 (20.0%)	3 (2.0%)	23 (16.5%)
Missed medical visit due to lack of transportation (past 12 months)?										
Yes	202 (23.5%)	32 (19.8%)	2 (8.7%)	23 (22.1%)	17 (14.2%)	16 (29.6%)	15 (32.6%)	14 (23.3%)	33 (22.0%)	50 (36.0%)
Never/rarely	515 (60.0%)	104 (64.2%)	18 (78.3%)	60 (57.7%)	82 (68.3%)	31 (57.4%)	27 (58.7%)	24 (40.0%)	105 (70.0%)	64 (46.0%)
No response	141 (16.4%)	26 (16.0%)	3 (13.0%)	21 (20.2%)	21 (17.5%)	7 (13.0%)	4 (8.7%)	22 (36.7%)	12 (8.0%)	25 (18.0%)
Significant depression?										
Yes	449 (52.3%)	83 (51.2%)	7 (30.4%)	43 (41.3%)	68 (56.7%)	30 (55.6%)	25 (54.3%)	23 (38.3%)	82 (54.7%)	88 (63.3%)
No	309 (36.0%)	61 (37.7%)	14 (60.9%)	38 (36.5%)	39 (32.5%)	17 (31.5%)	19 (41.3%)	20 (33.3%)	64 (42.7%)	37 (26.6%)
No response	100 (11.7%)	18 (11.1%)	2 (8.7%)	23 (22.1%)	13 (10.8%)	7 (13.0%)	2 (4.3%)	17 (28.3%)	4 (2.7%)	14 (10.1%)
Disclosed transgender identity?										
Yes	576 (67.1%)	104 (64.2%)	18 (78.3%)	53 (51.0%)	87 (72.5%)	34 (63.0%)	35 (76.1%)	45 (75.0%)	122 (81.3%)	78 (56.1%)
No	131 (15.3%)	29 (17.9%)	1 (4.3%)	20 (19.2%)	15 (12.5%)	7 (13.0%)	4 (8.7%)	5 (8.3%)	13 (8.7%)	37 (26.6%)
No response	151 (17.6%)	29 (17.9%)	4 (17.4%)	31 (29.8%)	18 (15.0%)	13 (24.1%)	7 (15.2%)	10 (16.7%)	15 (10.0%)	24 (17.3%)
Disclosed HIV status?										
Yes	536 (62.5%)	95 (58.6%)	13 (56.5%)	55 (52.9%)	78 (65.0%)	34 (63.0%)	27 (58.7%)	41 (68.3%)	112 (74.7%)	81 (58.3%)
No	183 (21.3%)	39 (24.1%)	5 (21.7%)	22 (21.2%)	23 (19.2%)	9 (16.7%)	12 (26.1%)	7 (11.7%)	31 (20.7%)	35 (25.2%)
No response	139 (16.2%)	28 (17.3%)	5 (21.7%)	27 (26.0%)	19 (15.8%)	11 (20.4%)	7 (15.2%)	12 (20.0%)	7 (4.7%)	23 (16.5%)
Taken hormones (past 6 months)?										
Yes	397 (46.3%)	63 (38.9%)	16 (69.6%)	58 (55.8%)	49 (40.8%)	30 (55.6%)	25 (54.3%)	27 (45.0%)	78 (52.0%)	51 (36.7%)
No	383 (44.6%)	91 (56.2%)	7 (30.4%)	32 (30.8%)	59 (49.2%)	18 (33.3%)	18 (39.1%)	15 (25.0%)	67 (44.7%)	76 (54.7%)
No response	78 (9.1%)	8 (4.9%)		14 (13.5%)	12 (10.0%)	6 (11.1%)	3 (6.5%)	18 (30.0%)	5 (3.3%)	12 (8.6%)
Employment discrimination (past 6 months)?										
Yes	302 (35.2%)	80 (49.4%)	9 (39.1%)	24 (23.1%)	36 (30.0%)	14 (25.9%)	10 (21.7%)	16 (26.7%)	75 (50.0%)	38 (27.3%)
No	394 (45.9%)	52 (32.1%)	12 (52.2%)	48 (46.2%)	63 (52.5%)	28 (51.9%)	30 (65.2%)	21 (35.0%)	65 (43.3%)	75 (54.0%)
No response	162 (18.9%)	30 (18.5%)	2 (8.7%)	32 (30.8%)	21 (17.5%)	12 (22.2%)	6 (13.0%)	23 (38.3%)	10 (6.7%)	26 (18.7%)
Shelter discrimination (past 6 months)?										
Yes	283 (33.0%)	72 (44.4%)	6 (26.1%)	20 (19.2%)	38 (31.7%)	21 (38.9%)	10 (21.7%)	24 (40.0%)	41 (27.3%)	51 (36.7%)
No	418 (48.7%)	60 (37.0%)	16 (69.6%)	56 (53.8%)	64 (53.3%)	24 (44.4%)	31 (67.4%)	16 (26.7%)	85 (56.7%)	66 (47.5%)
No response	157 (18.3%)	30 (18.5%)	1 (4.3%)	28 (26.9%)	18 (15.0%)	9 (16.7%)	5 (10.9%)	20 (33.3%)	24 (16.0%)	22 (15.8%)
Ever experienced transphobia?										
Yes	745 (86.8%)	150 (92.6%)	18 (78.3%)	84 (80.8%)	100 (83.3%)	47 (87.0%)	41 (89.1%)	45 (75.0%)	145 (96.7%)	115 (82.7%)
No	50 (5.8%)	3 (1.9%)	4 (17.4%)	7 (6.7%)	11 (9.2%)	4 (7.4%)	3 (6.5%)		4 (2.7%)	14 (10.1%)
No response	63 (7.3%)	9 (5.6%)	1 (4.3%)	13 (12.5%)	9 (7.5%)	3 (5.6%)	2 (4.3%)	15 (25.0%)	1 (0.7%)	10 (7.2%)
	Median (IQR)	Median (IQR)	Median (IQR)	Median (IQR)	Median (IQR)	Median (IQR)	Median (IQR)	Median (IQR)	Median (IQR)	Median (IQR)
Age	36 (28–46)	36.5 (30–47)	24 (22–26)	31 (26.5–42.5)	27.5 (23–35)	41.5 (31–51)	36.5 (29–44)	41 (32.5–50)	44 (38–50)	35 (28–44)
Healthcare empowerment	4 (3.75–4.75)	4 (4–5)	4.25 (3.75–4.75)	4 (3.63–5)	4 (3.75–4.38)	4.13 (3–5)	4.25 (3.25–5)	3.88 (3.25–4.25)	4 (4–4.5)	4 (2.75–5)

## 3 RESULTS

### 3.1 Participant characteristics

At baseline, the 858 participants’ median age was 36 years (Table 2). Almost half of the participants identified as Hispanic, Latina or of Spanish origin, and 42% identified as Black, non‐Hispanic. Two of every five participants reported having less than a 12th‐grade education. Additional participant characteristics are discussed in the Supplementary Materials.

### 3.2 Intervention exposure

During the first 24 months of follow‐up, 79% of participants had at least one interaction with intervention staff (Table 3). Most sites had over 90% participation in intervention components. Interventions that had lower‐than‐average participation included Trans Life Care, Howard Brown, and Trans Access, which implemented system‐level interventions that provided comprehensive trans‐competent services. Thus, their participants often interacted with the existing care system to receive routine services (not recorded as intervention exposure), in addition to specified intervention activities (recorded as intervention exposure).

**Table 3:** Summary of intervention exposure

	Overall	TWEET	INFINI‐T	Howard Brown	Trans Life Care	Trans Access	Brandy Martell	Princess Project	Transactivate	Alexis Project
Total participants enrolled	858	162	23	104	120	54	46	60	150	139
Participants exposed to intervention	676	152	19	20	58	39	44	57	150	137
% Exposed to intervention	79%	94%	83%	19%	48%	72%	96%	95%	100%	99%
Duration of intervention exposure (in hours)—median (IQR)	6.2 (3–12)	5.17 (1.67–11.13)	68.75 (35.53–210.72)	3 (2–7)	9 (3–15.83)	17 (9.25–45)	10.83 (6.25–32.08)	4.58 (2.75–7.08)	7.33 (4.5–11.33)	3.93 (2.07–6.87)
% Exposed to each type of contact:										
Outreach	2.66					35.90				2.92
Individual session	79.88	80.92	100		1.72	94.87	36.36	100	100	100
Group session	26.33	65.13	100	70		41.03	65.91	1.75		
Drop‐in session	13.17			40	98.28	48.72		8.77		
Virtual^a^	60.36	79.61	68.42		1.72	84.62	93.18	40.35	91.33	28.47
Median number of contacts (among participants with each type of contact):										
Outreach	1					1				1
Individual session	4	7	7		1	9	2	4	4	4
Group session	5.5	5	31	1		2.5	12	2		
Drop‐in session	3			2	3	6		1		
Virtual interaction^a^	3	1	10		16	5	34	1	7	2

^a^ Telephone call, text message, email, social media and postal mail.

Abbreviation: IQR, interquartile range.

The interventions provided substantial support to participants. Participants received services for a median of over 6 hours (interquartile range = 3–12 hours) over 24 months. Median time receiving services ranged from 3 hours at Howard Brown to 69 hours (almost 3 hours per month) in INFINI‐T. The majority of participants had in‐person individual sessions (80%; median = 4 sessions) and/or virtual sessions (60%; median = 3 sessions), including reminder phone calls. Fewer participants were reached through group sessions (26%; median = 5.5 sessions), drop‐in settings (13%; median = 3 visits) and outreach (3%; median = 1 contact).

### 3.3 Overall outcomes

**Figure 1:**
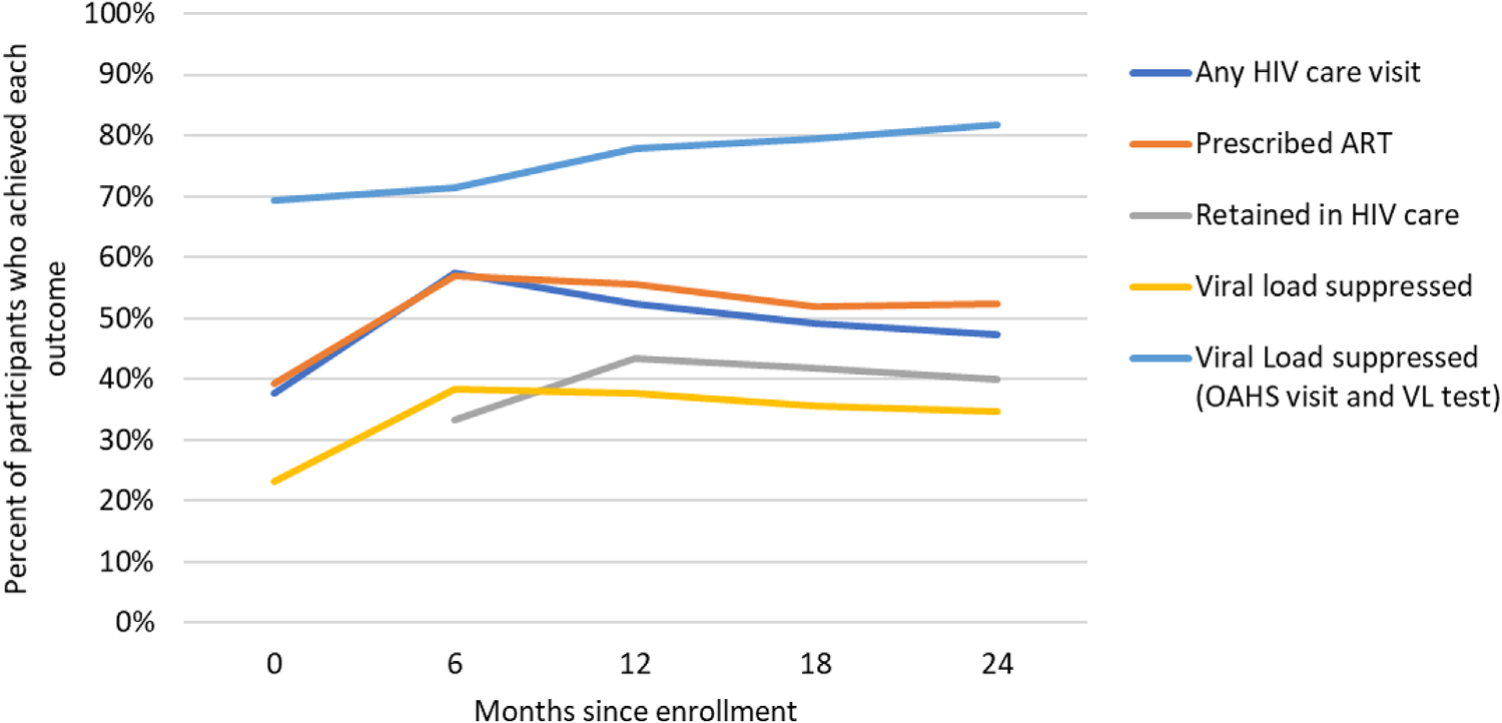
Changes in outcomes over time. Abbreviations: ART, antiretroviral therapy; OAHS, outpatient ambulatory health services; VL, viral load.

Figure 1 displays outcomes across all sites by follow‐up period. The proportion of participants who had a suppressed viral load increased from 22% at baseline to 38% at 6 months and decreased to 35% at 24 months. Trends were similar for any HIV care visit (38% at study entry, 58% at 6 months and 47% at 24 months), prescription of ART (39% at study entry, 57% at 6 months and 53% at 24 months) and retention in HIV care (33% at 6 months, 44% at 12 months and 40% at 24 months). In contrast, among participants who had an OAHS visit in addition to a viral load test during a study period, the proportion with suppressed viral load increased during each study period from 69% at study entry to 82% at 24 months.

The GEE models revealed that these increases for the overall sample were statistically significant at 12 months for all outcomes (unadjusted odds ratio [OR] = 1.38, 95% CI = 1.10–1.74 for any HIV care visit; OR = 1.42, 95% CI = 1.07–1.89 for prescription of ART; OR = 1.58, 95% CI = 1.03–2.42 for retention in HIV care; OR = 1.49, 95% CI = 1.23–1.81 for VS among all participants; and OR = 1.53, 95% CI = 1.23–1.89 for VS among those with an OAHS visit and viral load test) (Table 4). At 24 months, the increases remained significant for prescription of ART (OR = 1.49, 95% CI = 1.02–2.18), VS among all participants (OR = 1.54, 95% CI = 1.24–1.93); and VS among those with an OAHS visit and viral load test (OR = 1.98, 95% CI = 1.61–2.43).

**Table 4:** Unadjusted primary outcomes

	At 12 months follow‐up					At 24 months follow‐up				
	Any visit	Prescribed ART	Retained in HIV care	Suppressed viral load	Suppressed viral load (among those with OAHS visit)	Any visit	Prescribed ART	Retained in HIV care	Suppressed viral load	Suppressed viral load (among those with OAHS visit)
Overall sample	**1.38 (1.10, 1.74)**	**1.42 (1.07, 1.89)**	**1.58 (1.03, 2.42)**	**1.49 (1.23, 1.81)**	**1.53 (1.23, 1.89)**	1.34 (0.93, 1.92)	**1.49 (1.02, 2.18)**	1.73 (0.91, 3.32)	**1.54 (1.24, 1.93)**	**1.98 (1.61, 2.43)**
TWEET	**3.62 (1.54, 8.50)**	**3.02 (2.11, 4.31)**	**5.42 (2.78, 10.57)**	**2.67 (1.90, 3.75)**	2.03 (0.29, 14.05)	**3.85 (1.30, 11.40)**	**3.91 (2.49, 6.13)**	**11.28 (4.78, 26.65)**	**2.51 (1.64, 3.85)**	4.47 (0.53, 37.60)
INFINI‐T	**1.49 (1.15, 1.93)**	1.53 (0.65, 3.57)	**11.93 (2.58, 55.20)**	**2.39 (1.03, 5.51)**	1.00 (0.53, 1.89)	**1.52 (1.08, 2.13)**	1.28 (0.40, 4.10)	**24.98 (3.22, 193.47)**	2.38 (0.77, 7.34)	1.46 (0.63, 3.39)
Howard Brown	**1.81 (1.12, 2.96)**	1.30 (0.78, 2.18)	1.15 (0.58, 2.26)	**1.76 (1.24, 2.50)**	**2.09 (1.00, 4.34)**	1.87 (0.96, 3.65)	**2.57 (1.34, 4.93)**	1.06 (0.41, 2.71)	**2.01 (1.21, 3.35)**	**2.90 (1.11, 7.58)**
Trans Life Care	1.23 (0.83, 1.83)	1.07 (0.80, 1.40)	0.85 (0.49, 1.48)	0.96 (0.71, 1.28)	**1.93 (1.09, 3.41)**	1.12 (0.64, 1.94)	1.15 (0.82, 1.61)	0.76 (0.37, 1.57)	1.12 (0.75, 1.68)	**2.41 (1.16, 5.00)**
Trans Access	1.03 (0.69, 1.54)	**2.52 (1.50, 4.23)**	1.73 (0.80, 3.74)	**1.87 (1.11, 3.16)**	**0.50 (0.17, 1.50)**	0.71 (0.39, 1.30)	**2.87 (1.49, 5.53)**	2.05 (0.72, 5.85)	**2.16 (1.12, 4.19)**	0.42 (0.13, 1.43)
Brandy Martell	1.28 (0.98, 1.68)	1.20 (0.82, 1.74)	1.07 (0.54, 2.12)	1.23 (0.81, 1.87)	1.87 (0.98, 3.54)	1.08 (0.76, 1.53)	**1.59 (1.02, 2.47)**	1.03 (0.39, 2.69)	1.32 (0.73, 2.40)	2.19 (0.95, 5.08)
Princess Project	1.02 (0.66, 1.59)	0.98 (0.69, 1.41)	1.16 (0.54, 2.50)	1.03 (0.62, 1.73)	1.82 (0.76, 4.35)	0.87 (0.47, 1.60)	0.73 (0.41, 1.30)	0.86 (0.29, 2.57)	0.70 (0.34, 1.45)	**3.10 (1.19, 8.07)**
Transactivate	**2.31 (1.64, 3.24)**	**1.60 (1.22, 2.10)**	**1.70 (1.08, 2.68)**	**1.43 (1.09, 1.87)**	2.27 (0.89, 5.83)	**3.23 (2.06, 5.04)**	**1.48 (1.05, 2.09)**	**2.07 (1.14, 3.77)**	**1.56 (1.07, 2.26)**	2.87 (0.85, 9.66)
Alexis Project	0.90 (0.66, 1.24)	**1.33 (1.00, 1.77)**	1.23 (0.74, 2.04)	**1.61 (1.09, 2.39)**	1.01 (0.53, 1.95)	0.82 (0.54, 1.24)	1.15 (0.80, 1.64)	1.09 (0.58, 2.02)	1.43 (0.87, 2.45)	1.58 (0.69, 3.63)

Note: Bold indicates statistically significant result (*p*<0.05).

Abbreviation: ART, antiretroviral therapy; OAHS, outpatient ambulatory health services.

### 3.4 Outcomes by site

Table 4 also includes results from the unadjusted models for each site. We observed statistically significant (*p* < 0.05) increases in the odds of suppressed viral load at six of the nine sites at 12 months (Transactivate, TWEET, Alexis Project, INFINI‐T, Howard Brown and Trans Access) and at four sites at 24 months (Transactivate, TWEET, Howard Brown and Trans Access). Sites where the odds of any HIV primary care visit, prescription of ART or retention in HIV primary care were significantly higher at 12 months relative to baseline, also demonstrated increased odds of VS at 12 months. Multiple sites had higher odds of any HIV visit, prescription of ART or retention in HIV care at 24 months. Of the six sites where the odds of ART prescription at 24 months were significantly higher compared to baseline, five also had significantly higher odds of VS at 24 months.

Next, we employed inverse probability weighting to examine the effect of each site's intervention on HIV care continuum outcomes, while accounting for differences in client characteristics at baseline (see Supplementary Materials). While these models had larger confidence intervals than our unadjusted model due to increased variance associated with the estimation of weights and missing data, the estimates of effect (odds ratios) were similar or larger for five of the six sites where the odds of VS increased significantly between baseline and 12 months. The odds ratio for TWEET declined 15% (from 2.67 to 2.32), but this estimate of effect remained larger than that of any other site. We observed substantially more variability in the estimates of effect for odds of VS at 24 months, likely due to differential loss‐to‐follow‐up among clients who were less likely to remain engaged in HIV care.

## 4 DISCUSSION

TWC with HIV experience many direct and indirect barriers to care. The nine US‐based community service providers participating in this initiative developed strategies to help TWC overcome these barriers and engage in HIV‐related healthcare. Together, these clinics and community‐based organizations enrolled 858 TWC with HIV, of whom over three‐quarters received intervention services. Despite TWC often being reluctant to engage in health‐related programmes due to prior negative experiences with healthcare agencies, these organizations had great success recruiting participants and partnering with their local communities. Engaging trans women in leadership, staffing, and other meaningful roles has consistently been found to positively impact enrolment and retention in trans‐specific programming [48]. These strategies were successfully employed by most of these interventions to leverage social networks, increase trust, and provide peer navigation services that maximized participants’ safety and support.

Overall, the interventions achieved improvements in engagement in HIV care and VS at 12 and 24 months. Specifically, the odds of being virally suppressed at 12 and 24 months were significantly higher compared to baseline among all enrolled participants, among participants with an OAHS visit in the previous 6 months, and among participants in multiple sites (i.e. for six sites at 12 months and four sites at 24 months for VS among enrolled participants regardless of OAHS visit, and three sites each at 12 and 24 months for VS among those with an OAHS visit in the previous 6 months). This is an important success of the initiative. Of note, this improvement in VS was not always accompanied by a statistically significant improvement in retention in care suggesting that some intervention participants continued to take ART and achieved or maintained VS even if they did not always have the number and timing of appointments necessary to meet HRSA's definition of retention in care [46].

It is not surprising that sustaining engagement and retention in HIV care over 24 months was challenging since TWC with HIV experience intersectional oppression in the forms of racism and transphobia, community and institutional violence, stigma, discrimination, and marginalization from mainstream economic opportunities, which combine to adversely impact their access to healthcare and health outcomes [49]. Additional implementation research is necessary to study how interventions can be integrated into practice and maintained at the level of intensity necessary to result in sustained improvement in healthcare engagement and health outcomes over longer periods of time.

Given the challenges faced by TWC with HIV, the successes of these programmes demonstrate how well tailored they were to meet the specific needs of their communities. They are also a testament to the dedication of the intervention staff (many of whom were TWC), and the trusting relationships that developed between staff and participants [50]. While these programmes were heterogeneous in many ways, many shared important common elements. Recruiting, supporting, and retaining peer staff in HIV programming has consistently been found to contribute to positive outcomes [48]. In addition, many of these programmes aimed to address TWC's social determinants of health and intersecting vulnerabilities, including structural barriers, such as assistance with housing, legal concerns, and employment issues, and they provided trans‐affirming HIV care either on site or by referral. Indeed, the literature around health disparities increasingly acknowledges that it is critical that interventions to improve HIV‐related health outcomes among marginalized populations address social and structural factors that contribute to HIV‐related health disparities, rather than simply focus on individual‐level behaviour change [51, 52].

Due to the variations in the design and implementation of the individual interventions, we cannot pinpoint with certainty the intervention components that contributed to improvements in HIV care. However, we believe that keys to improving VS at 24 months included having structured activities clearly defined in written intervention curricula (e.g. TWEET), creating systemic change at the organizational level to make the entire system of care responsive to the needs of TWC with HIV (e.g. Howard Brown) and offering ongoing peer navigation/case management services to facilitate linkage to and retention in care (e.g. Trans Access and Transactivate). Some interventions did not last for 24 months (e.g. the Alexis Project); thus, VS not always being sustained beyond 12 months is not surprising.

### 4.1 Limitations

These findings should be interpreted in the context of certain limitations. First, the interventions were implemented in urban settings in the United States with participants whose median age was 36 years. Results may differ in programmes operating in dissimilar settings or with younger or older populations. Second, there were inconsistencies in how demonstration sites recorded various intervention activities (e.g. not all sites recorded short, virtual interactions). Therefore, we are limited in our ability to compare the effect of intervention exposure across interventions. Third, there was variability in the completeness of medical record data submitted for analysis. Thus, these results may underestimate the effect of some interventions. Fourth, one site enrolled few participants (INFINI‐T). This limited our ability to evaluate the effect of this intervention in larger settings. Fifth, these interventions were designed to “float above” the existing RWHAP services that the organizations already provided. A new activity that was integral to intervention at one site might have been routinely provided at another site. In the latter instance, these existing services were not recorded as exposure to the intervention being studied, thus making direct comparisons between the interventions difficult. Sixth, the structure of the HRSA SPNS Initiative prevented a randomized controlled trial design, which is used to develop evidence‐based interventions (EBIs). In addition, because of this community's great need for support, it was not viable to allocate some TWC to wait to receive these interventions. Thus, we are not able to determine whether broader changes in practice patterns or interventions external to this initiative contributed to the observed changes in engagement in HIV care. However, given the consistency of the results observed across the interventions, we are confident that these interventions enhanced engagement in HIV care among the TWC that they served.

## 5 CONCLUSIONS

Based on these findings, all the evaluated interventions meet HRSA's criteria for evidence‐informed interventions since they were based on theory and demonstrated improvement in at least one aspect of HIV care and treatment [35]. The evaluation results from this “real world” initiative are very promising since they documented significant improvements in engagement and retention in HIV care, ART prescriptions, and VS. Scientifically rigorous studies, such as adaptive trials or hybrid implementation science designs, could be conducted in the future on these or similar intervention models to help increase the number of EBIs that can further reduce HIV‐related health disparities. This HRSA SPNS initiative presents an alternative and complementary approach to the randomized‐controlled trial model to help ameliorate the research‐to‐practice gap and reduce health disparities among TWC with HIV. This approach is vital to eliminate inequalities that fuel the HIV/AIDS epidemic and ultimately achieve the UNAIDS 95‐95‐95 targets [53].

## COMPETING INTERESTS

The authors declare that they have no competing interests.

## AUTHORS’ CONTRIBUTIONS

All authors have read and approved the final manuscript. GMR, SBS, AM, JS and JGK made substantial contributions to conceptualizing and designing the national evaluation protocol. JMX conceptualized and designed the multisite initiative. SBS and DC analysed the data. GMR, DC, JMX, JS, AM and SBS wrote the paper.

## FUNDING

This project was supported by the Health Resources and Services Administration (HRSA) of the US Department of Health and Human Services (HHS) under grant number U90HA24973 in the amount of $536,244 awarded to the University of California at San Francisco.

## DISCLAIMER

The views expressed in this publication are solely the opinions of the authors and do not necessarily reflect the official policies of the US Department of Health and Human Services or the Health Resources and Services Administration, nor does mention of the department or agency names imply endorsement by the US Government. This publication was also made possible with help from the UCSF‐Center for AIDS Prevention Studies (P30 MH062246) and the UCSF‐Gladstone Center for AIDS Research (CFAR), an NIH‐funded programme (P30 AI027763).

## Supporting information


**Supplemental Table 1**. Adjusted Primary Outcomes.

## Data Availability

The data that support the findings of this study are available on reasonable request from the corresponding author. The data are not publicly available due to privacy or ethical restrictions.
