# Supplementary material for: An evaluation of nine culturally tailored interventions designed to enhance engagement in HIV care among transgender women of colour in the United States
Source: J Int AIDS Soc. 2022 Oct 12;25(Suppl 5):e25991. doi: 10.1002/jia2.25991 (PMC9557010; doi:10.1002/jia2.25991)
Supplement: Supplementary file 1 — Supplemental Table 1. Adjusted Primary Outcomes. [file JIA2-25-e25991-s001.docx]

**SUPPLEMENTAL MATERIALS**

Table 2 includes participant characteristics at study entry. Overall, median participant age was 36 years. Almost half the participants identified as Hispanic, Latina or of Spanish origin (49%), while over two of five identified as Black, non-Hispanic. Two of five participants reported having less than a 12^th^ grade education, while a third completed 12^th^ care and over two of five reported at least some college. Participants reported many life challenges. Over the past 6 months, two-thirds of participants reported running out of money for basics, two of five reported homelessness, almost two of five reported having exchanged sex to pay for necessities and one in ten reported having been incarcerated. In the past 12 months, two in five participants reported having missed a medical visit due to lack of transportation, over half reported significant symptoms of depression. Many participants reported stigma and discrimination. Over 8 in 10 participants reported ever having experienced transphobia, while one in three reported discrimination in employment and one in three reported discrimination in shelter in the past 6 months. In spite of these challenges, participants reported high levels of healthcare empowerment; almost half had received gender-confirming hormones in the past 6 months; two-thirds had disclosed their transgender identity and six in ten had disclosed their HIV status.

Participant characteristics varied substantially by site. Median age ranged between 24 years among HEAT Program participants and 44 years among Transactivate participants. The proportion of participants who reported Hispanic, Latino or Spanish origin ranged between 5% among Trans Life Care participants and 100% among Transactivate participants. Challenges meeting basic needs (having reported running out of money for basics, homelessness, and having exchanged sex to pay for necessities) varied between 22% and 77%, and incarceration in the past 6 months varied between 2% and 24%. Medical challenges (having missed a medical visit due to lack of transportation and having reported significant symptoms of depression) varied between 8% and 63%. Three in four participants within all sites reported ever having experienced transphobia while discrimination in employment or shelter varied between 22% and 55%. Healthcare empowerment was high across all sites (median ranged between 3.88 and 4.25), however, receipt of gender-confirming hormones varied between 37% and 56%, while disclosure of transgender identity and HIV status varied between 51% and 81%.

**Supplemental Table. Adjusted Primary Outcomes**

|  | **At 12 months follow-up** | | | | |  | **At 24 months follow-up** | | | | |
| --- | --- | --- | --- | --- | --- | --- | --- | --- | --- | --- | --- |
|  | **Any Visit** | **Prescribed ART** | **Retained in HIV Care** | **Suppressed Viral Load** | **Suppressed Viral Load (among those with OAHS visit)** ^†^ |  | **Any Visit** | **Prescribed ART** | **Retained in HIV Care** | **Suppressed Viral Load** | **Suppressed Viral Load (among those with OAHS visit)** ^†^ |
| **Overall Sample** | **1.89 (1.50, 2.39)**^§^ | **1.96 (1.35, 2.85)** | **1.97 (1.35, 2.89)** | **1.78 (1.32, 2.41)** | **1.35 (1.01, 1.79)** |  | 1.26 (0.84, 1.89) | 1.38 (0.94, 2.04) | **1.39 (0.95, 2.05)** | **1.40 (1.11, 1.78)** | **2.58 (1.87, 3.58)** |
| **TWEET** | **2.34 (1.00, 5.46)** | **3.05 (1.22, 7.60)** | **15.29 (3.08, 78.24)** | 2.32 (0.90, 5.98) | 1.58 (0.34, 7.40) |  | 1.91 (0.82, 4.44) | 2.07 (0.94, 4.58) | **18.24 (3.30, 101.61)** | 1.39 (0.59, 3.26) | 2.96 (0.49, 17.95) |
| **INFINI-T** | **64.57 (1.25, 10201)** | 1.82 (0.94, 4.24) | **199665 (16.27, 4573385254)** | 6560.06 (0.86, 155193893) | **16942166 (3930.71, 74548553111)** |  | 12.84 (0.39, 521.47) | 0.14 (0.04, 0.72) | **331385 (24.16, 8045487692)** | 11492 (0.99, 282914547) | **17465217 (5203.02, 59698116254)** |
| **Howard Brown** | **1.88 (0.88, 4.02)** | 0.82 (0.68, 0.99) | 2.99 (0.67, 13.41) | **2.20 (1.29, 3.75)** | 2.16 (0.74, 6.32) |  | 1.13 (0.43, 2.96) | **4.63 (2.34, 9.19)** | 2.73 (0.65, 11.52) | **2.62 (1.03, 6.68)** | **4.37 (1.24, 15.52)** |
| **Trans Life Care** | 1.19 (0.87, 1.63) | 1.21 (0.85, 1.72) | 1.51 (0.76, 3.01) | 0.87 (0.38, 1.99) | 0.91 (0.28, 2.96) |  | 0.74 (0.45, 1.21) | 1.01 (0.68, 1.51) | 1.11 (0.57, 2.15) | 1.09 (0.70, 1.70) | 2.11 (0.67, 6.60) |
| **Trans Access** | 2.42 (0.94, 6.21) | 2.37 (0.77, 7.33) | 4.73 (0.69, 33.03) | 1.97 (0.55, 7.14) | 2.17 (0.55, 8.59) |  | 1.03 (0.31, 3.52) | 1.85 (0.49, 7.20) | 2.54 (0.25, 26.98) | 1.26 (0.34, 4.74) | 2.15 (0.49, 9.51) |
| **Brandy Martell** | 1.82 (0.85, 3.90) | 1.49 (0.93, 2.41) | 1.45 (0.15, 14.30) | 1.67 (0.95, 2.94) | 1.87 (0.65, 5.42) |  | 0.77 (0.44, 1.35) | 1.49 (0.96, 2.31) | 1.24 (0.21, 7.23) | 1.03 (0.47, 2.24) | 2.74 (0.76, 9.12) |
| **Princess Project** | 0.84 (0.62, 1.15) | 1.08 (0.65, 1.80) | 0.80 (0.32, 2.02) | 0.57 (0.18, 1.83) | 0.05 (0.00, 0.66) |  | 0.18 (0.05, 0.65) | 0.26 (0.07, 0.95) | 0.18 (0.04, 0.85) | 0.15 (0.03, 0.77) | 0.02 (0.00, 0.36) |
| **Transactivate** | **1.87 (1.26, 2.77)** | **2.24 (1.53, 3.27)** | 2.43 (0.88, 6.77) | **1.67 (1.03, 2.69)** | 0.77 (0.21, 2.87) |  | 1.37 (0.74, 2.55) | 1.32 (0.75, 2.33) | 2.52 (0.86, 7.37) | 1.32 (0.62, 2.83) | 2.17 (0.41, 11.45) |
| **Alexis Project** | **1.79 (1.30, 2.48)** | **1.69 (1.22, 2.34)** | **2.91 (1.37, 6.15)** | **1.95 (1.24, 3.06)** | 1.56 (0.74, 3.29) |  | 0.90 (0.57, 1.42) | 1.04 (0.65, 1.64) | 1.83 (0.90, 3.72) | 1.62 (0.80, 3.28) | 2.96 (0.98, 8.98) |

Note: All models reported in this table adjusted for participant characteristics at baseline (also listed in Table 2) – race-ethnicity (dichotomized as Hispanic vs. non-Hispanic), education (dichotomized as at least some college vs. no college), financial security, homelessness, transactional sex, incarceration, missed medical visits due to lack of transportation, depression, disclosure of transgender identity, disclosure of HIV status, intake of gender-confirming hormones, experience of discrimination in employment and shelter, lifetime experience of transphobia, age, and level of health care empowerment.

^§^ Bold indicates statistically significant result (p<0.05).

^†^ OAHS: Outpatient ambulatory health services
